# Supplementary material for: Are carbon emissions trading and green financial instruments synergistic? -Comprehensive quantitative research based on content analysis
Source: PLoS One. 2024 Mar 7;19(3):e0298601. doi: 10.1371/journal.pone.0298601 (PMC10919601; doi:10.1371/journal.pone.0298601)
Supplement: S1 Data — (ZIP) [file pone.0298601.s001.zip › Data/地级市Z_F工作报告.zip!/╔┬╬≈/╧╠╤⌠╩╨/└┤╫╘╣1⁄2╓┌║┼í╛┬φ┐╦╩2╛▌═°í┐_2018.docx]

各位代表：

现在，我代表市人民政府向大会报告工作，请予审议，并请各位市政协委员和列席人员提出意见。

一、2017年工作回顾

2017年，在省委、省政府和市委的坚强领导下，全市上下以习近平新时代中国特色社会主义思想为指引，坚持稳中求进工作总基调，紧盯追赶超越目标，践行“五个扎实”要求，落实“五新”战略任务，埋头苦干，奋力攻坚，全市经济社会平稳发展，实现了新一届政府良好开局。

一年来，一批支撑发展、引领转型的产业项目取得重大突破。8.6代液晶面板项目点亮投产，正泰输配电产业加速聚集，秦星电动公交车投放市场，转型发展成效初显。

一年来，一批优化功能、提升品质的标志性工程建成投用。城市总体规划经省政府批复；渭河生态治理进展顺利，古渡廊桥建成通行，“一河两岸”成为都市会客厅；市民文化中心建成使用，双照湖如期蓄水，“三城两带”城市空间格局初步形成。

一年来，一批群众期盼、社会关注的民生实事扎实推进。成功创建全国文明城市，荣获全国未成年人思想道德建设工作先进城市称号。脱贫攻坚超额完成全年任务。30件惠民便民实事完成投资101亿元。7个县区顺利通过全国义务教育发展基本均衡县验收，提前两年实现全覆盖。

各位代表，这些重大工程、民生大事，久久为功、方成始终。我们将始终保持善谋事、干成事的韧劲和拼劲，不断迈出追赶超越的铿锵步伐！

一年来，重点抓了以下工作：

一是有力应对多重挑战，全市经济总体企稳。积极适应西咸新区体制调整带来的新变化，主动应对铁腕治霾等工作带来的新挑战，着力解决经济深度调整产生的新问题，统筹兼顾、精准施策，强化工业抓项目，培育新兴调结构，发展县域强支撑，补齐短板惠民生，全市经济平稳增长、质量提升。全市生产总值达到2340.65亿元、增长8.1%；地方一般公共预算收入完成77.5亿元、同口径增长15.2%，税收占比达到77.5%，财政支出346.5亿元、增长11.3%；城乡居民人均可支配收入分别达到34246元、11430元，增长8.2%、9.1%。县域经济平均规模达到147.87亿元，比2016年增加8.6亿元；咸阳高新区综合实力在全国排名97位，较上年提升27位。

二是突出工业加快转型，产业结构优化升级。实施工业发展“3+1111”工程，乾县优利士、旬邑海天制药等36个工业重点项目建成投产，工业投资增长7.8%，新增规模以上工业企业62户。康惠制药成功上市，宝塔山油漆“新三板”挂牌，陕西股权交易中心挂牌企业达到153家。规模以上工业企业利润总额增速同比提高2.4个百分点，非能化产业增速高出能化产业7.5个百分点。全市规模以上工业总产值、增加值分别达到3197.21亿元、1053.59亿元，增长15.2%、8.5%。农业供给侧结构性改革取得新进展，新发展矮化果园6.01万亩、苗木花卉15.09万亩，农民专业合作社、家庭农场分别达到4671个、1251个，“咸阳马栏红”苹果商标正式启用。全年粮食、水果、蔬菜和肉蛋奶总产分别达到163.25万吨、574.85万吨、361.82万吨、91.94万吨。现代物流、电子商务保持高速增长，三原、旬邑、淳化成功创建国家级电子商务进农村综合示范县，武功荣获全国电子商务创新发展百强区县和“互联网+农业”十大标杆县。全市旅游综合收入达到292.2亿元、增长27%，郑国渠旅游景区、云集生态园景区被评为4A级景区。共享经济、移动支付等新业态加速发展。社会消费品零售总额达到613.04亿元、增长14%。

三是强化项目带动作用，新的增长点加速形成。全社会固定资产投资2382.67亿元、增长11.3%，高技术产业、战略性新兴产业投资分别增长75.5%、60.3%。145个市级重点项目累计完成投资613.6亿元，占年计划的103.4%。总投资390亿元的19户液晶面板上下游企业加快建设，电子信息产业振兴势头迅猛。启动装备制造产业园建设，正泰电气5条产品线搬迁来咸，聚集配套企业15户，输配电产业加速发展。秦星大中型纯电动车和专用车生产线项目加快建设，我市成为全省新能源汽车产业布局重点板块。招商引资引进合同项目274个，实现到位资金651.5亿元；实际利用外资7678万美元、增长2.82倍，增速位居全省第二。与高校及科研院所产学研合作不断深化，新增国家及省级科技企业孵化器4家、省级众创空间2家，培育瞪羚企业30户、科技小巨人企业28户、高新技术企业18户，省质量名牌达到74个。技术合同交易额和发明专利申请量分别完成年度任务的140.7%、126.6%。

四是加强基础设施建设，城乡融合不断深化。坚决落实省委、省政府西咸新区体制划转决策，全面完成4个县市区15个街镇行政和社会管理职能托管移交，秦都区顺利承接渭河南城12.6平方公里行政和社会管理职能。以创文促城建，完成7条道路改造工程，修补主次干道128条，改造26条道路路灯，整治提升凤凰广场、火车站广场等12个广场。新投放公交车200辆，启动智慧公交电子站牌替代工作，我市入选全国公交都市创建城市。16个省市重点镇、文化旅游名镇完成投资14.5亿元，占年计划的110.4%，长武亭口镇入选第二批全国特色小镇。交通设施完成投资30亿元，其中，农村公路10.6亿元；水利设施完成投资17.3亿元，柏岭寺水库蓄水，张家山泉群供水工程通水，城乡基础设施通达程度进一步提升。

五是落实责任铁腕治霾，生态环保有力加强。投入财政资金6.66亿元，加大力度治霾治污，扎实开展“1+14”专项行动，全力减煤、控车、抑尘、治源、禁燃、增绿，全年PM10、PM2.5平均浓度同比分别下降8.7%、2.4%。加大曝光力度，健全长效机制，农村环境卫生面貌明显改善。全面推行“河长制”，城镇日污水处理能力达到76万吨，11个国省考监测断面水质全部达标。严明责任、时限和标准，中央和省委环保督察反馈问题整改工作进展顺利。启动国家森林城市创建工作，完成营造林38.2万亩，绿化道路326公里，淳化、旬邑成功创建国家园林县城。国家湿地公园达到6个，湿地保护面积13.3万亩。

六是精准实施脱贫攻坚，民生福祉持续增进。把脱贫攻坚作为头等大事、第一民生工程，财政专项扶贫资金达到8.01亿元。“八个一批”措施扎实推进，引进正大集团100万头生猪全产业链等一批产业扶贫项目；“三变”改革有序推进，率先在全省实现市县两级农业投融资担保业务全覆盖；易地扶贫搬迁5142户17890人，在全省率先完成“十三五”易地扶贫搬迁任务；建立“四重保障”制度，推行健康扶贫“微改革”，新农合累计为贫困群众报销费用3.3亿元；咸阳·泰州扶贫协作、中行定点扶贫以及“3+X”帮扶扎实推进，“公益中行”精准扶贫平台网上交易额累计超过9000万元，全年脱贫4.48万人，退出贫困村219个。着力办好民生实事，城镇新增就业5.43万人，城镇登记失业率控制在3.2%。教育基础设施共完成投资7.37亿元，英才学校主体封顶，彩虹第二中学加快建设，普惠性幼儿园占比达到68.1%。综合医改深入推进，公立医院药品加成全面取消。妥善处置H7N9疫情。省十六运筹备有序推进，15个涉赛场馆建设进展顺利。体育惠民工程及渭河全民健身长廊完成投资5633万元，成功举办渭河国际马拉松邀请赛。机关事业养老保险制度实现全覆盖，城乡居民跨省异地就医住院费用直接结算基本实现。解决了54.7万群众饮水不安全问题。棚户区改造开工项目22个、11581套，保障性住房分配入住14541套。“四反十打”成效显著，安全生产形势保持平稳，社会大局和谐稳定。

七是深化改革提升效能，发展环境持续改善。持续深化“放管服”改革，自主取消下放市级行政审批事项43项，第一批413项“最多跑一次”事项公布实施，彬县、泾阳“一枚印章管审批”及兴平“市民之家”“一厅式办理、限时化办结”试点顺利推进，公共资源交易平台建成投用。“减证便民”专项行动深入推进，“双随机一公开”监管模式实现全覆盖，规范市场秩序取得积极进展，我市被确定为西北首个网络市场监管与服务示范区创建市。国有资产管理体制进一步理顺，市属国有资产规模跨上千亿台阶。全面完成国有林场改革，农村承包地确权登记颁证、农村集体产权制度改革、供销社综合改革进展顺利。“三去一降一补”深入推进，30万吨/年以下煤矿全部关闭，房地产去库存周期缩短到9.6个月，全年为企业共减免税17.24亿元，为61户企业争取直供电17.35亿度。市场活力加速释放，新增各类市场主体32890户、增长27%，非公经济占比达到52.7%。自觉接受人大监督、政协监督和社会监督，扎实开展“行政效能提升年”活动，“四级联网、服务下沉”政务服务模式稳步推进，“五型”政府建设迈出新步伐。

双拥、老龄、残疾人、红十字、妇女儿童等事业取得了新进步，外事、侨务、对台、审计、统计、民族、宗教、地震、气象、人防、档案、盐务、地方志等工作实现了新发展。

各位代表，过去一年，在省委、省政府及市委坚强领导下，广大干部群众顾全大局、勠力同心、克难奋进，各项工作取得新成就；广大脱贫攻坚战线的干部职工心系群众、忘我工作、甘于奉献，广大贫困群众有了更多获得感。在此，我代表市人民政府，向全市广大干部群众，向各民主党派、人民团体，向驻咸人民解放军、武警消防官兵和政法干警，向关心支持咸阳发展的各界人士，表示衷心的感谢和崇高的敬意!

发展成绩是前进的基石，存在问题是改进的方向。我们清醒地认识到，发展不平衡不充分问题在各领域还不同程度存在，主要表现在：重大工业项目、重大基础设施项目接续不力，投资后劲不足，民间投资比较低迷。工业结构偏重，产业竞争力不强，发展方式比较粗放。生态环境质量不优，优质公共服务资源与群众需求有较大差距，“入园难”“看病贵”“停车难”“出行堵”等问题需要进一步加大力度解决。惰政怠政的问题还没有彻底根治，营商环境需要进一步优化。我们将直面问题，下决心解决问题，努力把咸阳的事情办好，决不辜负人民群众的期待！

二、2018年目标任务

2018年是贯彻党的十九大精神的开局之年，是改革开放40周年，是决胜全面建成小康社会、实施“十三五”规划承上启下的关键一年，第十六届省运会将在咸阳举办，做好今年工作意义十分重大。

我们将高举习近平新时代中国特色社会主义思想伟大旗帜，坚持稳中求进工作总基调，坚持新发展理念，以推动高质量发展为根本要求，以供给侧结构性改革为主线，深入践行“五个扎实”要求，全面落实“五新”战略任务，统筹推进稳增长、促改革、调结构、惠民生、防风险、强党建各项工作，善谋划、提质量、优营商、增效能，全力打好精准扶贫脱贫、生态环境保护、优化营商环境、巩固创文成果“四场攻坚战”，迈出新时代爬坡过坎、追赶超越更大步伐，取得建设富强人文健康新咸阳更大进展。

主要预期目标是：生产总值增长8.5%左右，规模以上工业增加值增长8.5%左右，全社会固定资产投资增长13%左右，社会消费品零售总额增长12%左右，地方一般公共预算收入同口径增长9%左右，城镇登记失业率控制在4%以内，CPI涨幅控制在3%左右，城镇居民人均可支配收入增长8.5%左右，农村居民人均可支配收入增长9%左右，单位GDP能耗下降3.2%左右。

在工作中，重点把握好以下几点：

一是坚持以人民为中心的发展思想。把为民惠民作为一切工作的发力点和着力点，坚决打好精准扶贫脱贫攻坚战，促进城乡居民收入增速快于经济增速。持续提升公共服务质量，加快优化生态环境，着力补齐民生短板，在增进民生福祉上不断取得新进展，满足人民日益增长的美好生活需要。

二是落实高质量发展的根本要求。主动适应新时代我国社会主要矛盾新变化，深入贯彻落实新发展理念，坚持质量第一、效率优先，扎实推进供给侧结构性改革，突出产业结构调整，推进系统转型；激活创新第一动力，推进质量变革、效率变革、动力变革，努力推进经济总量追赶、质量效率超越、发展模式创新，持续增强经济创新力和竞争力。

三是牢牢扭住项目谋划建设不放松。坚持经济工作项目化、项目工作责任化、落实责任制度化，以项目落实政策，以项目聚集要素。健全项目工作问责激励机制，加大项目谋划推进力度，提升招商引资精准化水平，以项目提质量、增后劲，以项目优生态、惠民生，以高质量项目推动高质量发展，以大项目助力追赶超越。

三、2018年重点工作

（一）调整产业结构，着力构建现代化经济体系

改造升级传统产业。坚持工业主导不动摇，扎实推进工业发展“3+1111”工程。加快神华CFB示范项目前期工作，推进30万吨乙二醇等项目建设，促进彬长旬高端能化基地加快发展。鼓励建材企业发展环保产业，推进建材产业绿色转型。开展“三品”专项行动，支持伊利西北乳制品生产基地、杜克普高端智能定制等项目建设，促进食品、纺织等产业技术创新、产品创新、业态创新。完善激励扶持政策，引导企业加大技改力度，促进技改投资稳定增长。加大“僵尸企业”处置力度，有效盘活土地、厂房等闲置资产，实现“腾笼换鸟”。

培育壮大新兴产业。抓手是招商，核心在项目，动力是创新，路径是融合。抢抓中省鼓励新能源汽车产业发展机遇，按照“一心一地多点”布局，加大市场份额和政策倾斜力度，支持秦星新能源汽车项目加快建设，积极引进龙头企业，扶持发展高精尖配套产业，努力把新能源汽车打造成千亿级产业集群。支持8.6代液晶面板上下游配套企业上半年实现量产，让液晶面板成为咸阳制造新名片。加快装备制造产业园发展，推进正泰上海松江园区整体搬迁，再引进一批高端装备制造龙头企业。以高新区医药产业园、旬邑中医药健康产业园为载体，发挥步长、修正、康惠等企业带动作用，把招商链、创新链、金融链、服务链建在产业链上，培育壮大生物医药产业。深入实施“互联网+”行动计划和“中国制造2025”，加大机器人、新材料以及生态环保等新兴产业培育力度，加快发展动能转换步伐。用足用好国家级高新区金字招牌，支持“两区两园”加快发展，打造新的经济增长极。

大力发展现代服务业。发展壮大现代物流，发挥中银西部物流基金撬动作用，启动西部物流产业园建设；健全城乡物流体系，推动冷链物流、多式联运融合发展。实施电子商务提升行动，支持电子商务示范创建，积极培育现代供应链，促进电子商务与一二三产融合渗透。推进全域旅游示范市创建，着力构建“两核两轴两带两廊道”旅游产业空间格局。提升乡村旅游品质，培育冰雪旅游、度假旅游、休闲旅游新业态。支持乾陵创建5A级景区，启动泾河户外运动公园建设。办好国庆渭河马拉松赛、国际风筝节，策划一批高端体育赛事，培育发展体育产业。实施商贸服务业提升行动，鼓励发展养老服务、共享经济、会展经济等服务业，优化服务业供给结构。

提升金融服务实体经济能力。增强市融资担保公司服务能力，推进省农担县级办事处加快运营。加快4个贫困县村镇银行筹建步伐，健全金融业服务体系。规范发展市县两级专业融资平台，密切银政企协作，引导银行业金融机构加大对地方经济支持力度。实行国有资产集中统一监管，确保国有资产保值增值，增强国有资本对培育支柱产业、重大基础设施建设、改善民生的撬动引导作用。加大企业上市培育力度，积极对接多层次资本市场，提高直接融资比重。引导各类金融机构与众创平台、孵化器、中小微企业联动发展，以金融产品创新服务企业发展壮大，以资本链催生产业链。严厉打击非法金融活动，优化金融生态环境。

（二）实施多轮驱动，着力推进质量变革效率变革动力变革

增强招商实效。严格招商工作责任，加强对县市区及部门主要领导招商实绩的督查考核。围绕打造产业集群，紧盯国内500强、民营100强及主板上市公司，借力中国银行定点扶贫、咸阳·泰州扶贫协作，发挥商会牵线搭桥和龙头企业带动作用，突出工业项目、重大项目，力求招一个、成一个，着力提升招商引资精准性实效性。细化项目落地引导机制，实行招商项目落实督办制度和重大招商项目招委会落实推进制度，力争全年招商引资到位资金700亿元。

强化项目支撑。实施投资“九大工程”，市级领导负责，全流程推进项目策划包装、招商引资及落地建设、环境保障等工作，形成谋项目、抓项目、上大项目的浓厚氛围。全力抓好年度投资481.99亿元的155个市级重点项目，加强考核督查，积极协调解决土地、审批等问题，促进雷达精确制导全天候巡检仪等63个续建项目建成投产、东方雨虹防水卷材及防水涂料生产等92个新建项目开工建设，加快锂电池产业链等74个前期项目落地开工。进一步提高PPP项目策划包装质量，吸引社会资本投向基础设施、公共服务等领域，促进民间投资回升。

加强创新引领。积极创建国家创新型试点城市，扎实推进国家知识产权试点城市建设。实施县域创新驱动发展示范工程，加强与西北大学、西安邮电大学等高校和科研院所合作，推动中医药大学、陕工职院、高新区等众创空间发展，深化创新链、资本链、产业链对接融合，加大瞪羚企业、科技小巨人企业、高新技术企业培育力度。实施重大人才工程，以人才带技术、引项目、促创新。深入实施质量强市及名牌战略，创造更多咸阳标准、咸阳品牌。深化军民融合、央地协作，实施军民融合产业创新发展工程，支持兴平、三原、武功创建省级军民融合示范基地。

（三）深度融入“一带一路”建设，着力推进枢纽经济门户经济流动经济发展

强化交通枢纽功能。围绕打造立体交通大通道，积极服务银西高铁及菏宝高速旬凤段、银百高速陕甘界至旬邑段2条高速公路建设，全力保障西安至阎良、机场至法门寺2条城际铁路建设，加快推进国道108三原至礼泉、国道344兴平至武功、省道107礼泉赵镇至乾县临平等11条国省干线公路工程建设，年内建成西咸北环线店张立交，新开工西宝高速老线沣河出口、新线兴平阜寨出口以及福银高速3个出口改扩建工程。积极谋划通用机场、货运机场、运煤专线和高速连接线项目，科学布局发展物流园区，着力构建陆空一体、多式联运交通物流体系。

打造内陆开放开发高地。认真落实关中协同创新发展战略，积极推进西咸一体化，争取地铁1号线三期、11号线获批开工。加快基础设施联通、公共服务均等、营商环境同质、发展资源共享步伐，合力共建大西安，服务陕西“一带一路”核心区建设。实施扩大对外开放行动，提升中韩产业园的影响力，吸引国内外知名企业来咸建设面向西部的生产加工基地、商贸物流基地，打造产业西进的承接地。发挥机场的门户功能，培育发展临空经济、跨境电商，带动制造业服务化、农业品牌化、服务业特色化发展。扩大国际产能合作，加快推进中哈产业园咸阳纺织工业园区建设，策划举办“丝绸之路文化艺术节”，搭建对外开放合作平台，提升经济外向度。

（四）实施乡村振兴战略，着力促进城乡融合发展

加快发展现代农业。坚持质量兴农、绿色兴农，发挥紧邻杨凌农科城优势，深化农业供给侧结构性改革，加快发展优质高效、生态观光现代农业。实施粮食安全战略，启动粮食功能区划定工作，推进高标准农田建设；引导苹果向优生区集中，提升蔬菜产业组织化品牌化水平，促进生猪、奶山羊全产业链发展，扶持旱腰带地区发展石榴、酿酒葡萄及酒庄产业，扩大苗木花卉、猕猴桃、食用菌、中药材等特色产业规模。加强农产品“三品一标”认证，支持农产品安全质量监管示范创建，提升“咸阳茯茶”“咸阳马栏红”“武功小子”“三原香油”等品牌的市场竞争力和产业链整合力。培育壮大新型农业经营主体，支持现代农业产业园区发展，积极争取田园综合体试点，带动一二三产融合发展，健全利益联结机制，让农民分享全产业链增资收益。发展智慧农机，提升农业机械化示范区建设水平。推进土地整治，改善农田灌溉设施，发展高效节水灌溉8万亩。

推进美丽乡村建设。加强乡村规划编制，行政村规划编制覆盖率达到80%以上。推动农村基础设施提档升级，按照“四好”农村公路要求，改造新建农村公路1600公里以上，3年内实现组组通；实施农村安全饮水巩固提升和自然村通动力电提升工程。继续开展农村人居环境整治行动，大力推进“厕所革命”，推广环境卫生整体外包模式，强化农业面源污染防治。开展农村风貌整治，保护传统村落，支持创建美丽宜居示范村，让农村生态美起来、环境靓起来。

深化农业农村改革。落实“第二轮土地承包到期后再延长三十年”政策，深化“三权分置”和农村集体产权制度改革，健全农村产权交易服务体系，吸引工商资本 “下乡”，激活农村沉睡的资源。建立实施乡村振兴战略领导责任制，夯实党政“一把手”责任。健全财政投入保障制度，以县为主推进涉农资金整合。加强“三农”工作队伍建设，实施新型职业农民培育工程。创新城乡融合发展体制机制，消除要素下乡障碍，让农村成为创新创业的新天地。

提升乡村治理能力。建立健全党委领导、政府负责、社会协同、公众参与、法治保障的治理体制和自治、法治、德治相结合的乡村治理体系，完成第十次村委会换届选举工作，提升村务公开制度化规范化水平。推广“枫桥经验”，推进平安乡村建设。加强农村精神文明建设，传承发展提升农村优秀传统文化；开展移风易俗行动，发挥村规民约导向作用，培育文明乡风、良好家风、淳朴民风、婚嫁新风。加强对农村留守儿童、留守妇女、留守老人的关爱和服务，加大“天价彩礼”、婚丧大操大办、封建迷信等群众反映突出问题的综合整治力度，让农村成为宜居幸福家园。

做大做强县域经济。抢抓建设国家级关中平原城市群的战略机遇，落实加快县域经济追赶超越的意见，引导各县市区发挥地处大西安经济圈的优势，不断强化工业主导，下好招商引资先手棋，做好“园区发展、民营经济、城镇建设”三篇文章，补齐“发展环境、公共服务”两个短板，在做强特色产业、推进城乡融合发展上创新路。支持彬县、三原撤县设市，支持泾阳开辟发展新空间、渭城发展以楼宇经济为重点的都市经济，引导秦兴武联手打造工业走廊、礼泉与大西安（咸阳）文体功能区联动发展，鼓励乾县做强“陕西研发、乾县配套”，加快彬长旬煤炭转化，提升淳化、永寿绿色发展能力。支持县域工业集中区创建省级示范园区，提升县域工业集中区基础设施承载能力和产业集群化水平。加强县城改造扩容提质和重点示范镇建设，鼓励发展特色小镇，带动城乡融合发展。

（五）以举办省十六运为契机，着力提升城市现代宜居水平

提速发展文体功能区，拓展城市新空间。推动基础设施建设大提速，建成锦秀山公园、暖温带森林博览园，道路主骨架全面形成。推动公共服务项目建设大提速，市民大厅、市特殊教育学校等民生项目启动建设，完成丝路大学城规划。推动产业发展大提速，红星美凯龙家居智慧物流城等5个现代物流项目加快建设，借势省十六运，再引进建设一批临空经济、会展经济、文化创意、体育休闲、现代物流项目，努力在发展枢纽经济门户经济流动经济上走在全省前列。

实施城建五大工程，塑造城市新品质。实施道路对接、疏堵保畅、品质提升、立体绿化、精细管理五大工程，重点抓好年度投资189亿元的156个城建项目。启动咸北大道建设工程，完成东风路、文林路等17条城市主干道路改造提升。扎实开展公交都市创建，再投放100辆新能源公交车，加快公交电子站牌替换步伐，建成人民路公交专用道，提升公交线路通达深度。建设5座人行天桥，规范管理共享自行车，加快停车场建设，多措并举疏堵保畅。实施金旭路、渭阳西路等片区雨污分流及基础设施提升改造工程，加快根治城市内涝。推行立体绿化，打造特色街区，建设一批市民身边的小绿地、小广场。启动渭河南城提升工作，加快咸阳湖景区提升、市体育场改造，全面建成渭河生态治理南岸工程，使“一城绿树半城湖”成为城市最靓丽的名片。

全力办好省十六运，彰显城市新形象。高质量推进奥体中心等涉赛场馆建设和改造提升工程，为广大运动员提供一流比赛场地。精心策划编排开闭幕式，扩大火炬传递的参与度和影响力，营造人人争当省运会文明使者的浓厚氛围，展现文明咸阳新形象。做好省运会期间水电气讯、交通保障、志愿者服务、安全保障等各方面工作，完善应急预案，办一届精彩纷呈、质量一流、彰显魅力、全民共享的体育盛会。

各位代表，办一届精彩的省运会，是承诺、是责任，也是咸阳难得的发展机遇。我们将以此为契机，促进各项工作上台阶，为高质量发展增动力，让“大秦故都、德善咸阳”城市品牌更靓丽！

（六）坚决打好“四场攻坚战”，着力补短板增优势

坚决打好精准扶贫脱贫攻坚战。坚持精准扶贫、精准脱贫，把提高脱贫质量放在首位，推进“八个一批”措施精准落实，确保长武、淳化和旬邑3个贫困县今年摘帽，442个贫困村达标退出，6.6万贫困人口稳定脱贫。把壮大产业扶贫作为重中之重，深化“三变”改革，支持市农投公司发展，加大龙头企业和合作经济组织培育力度，提升扶贫产业规模化组织化水平。以基础设施、公共服务及产业项目为重点，争取六盘山片区扶贫攻坚政策支持，对接中国银行定点扶贫，深化与泰州扶贫协作。全力抓好3个深度贫困村脱贫攻坚，确保发展条件明显改善、贫困群众收入增速明显加快。扶贫与扶志扶智相结合，激发贫困群众内生发展动力。严格落实“双包双沉”市县领导包抓责任，进一步提升“四支队伍”战斗力。加大扶贫领域腐败和作风问题专项治理，确保真扶贫、扶真贫、真脱贫。

坚决打好生态环境保护攻坚战。提高站位，严格责任，标本兼治，彻底整改中央和省委环保督察反馈问题。以必胜的决心、更大的力度推进铁腕治霾，制定新一轮保卫蓝天三年行动方案，下决心减煤、控车、抑尘、治源、禁燃、增绿，确保主要污染物大幅减少、空气质量持续好转。自然修复与生物措施、工程措施相结合，加快旱腰带地区生态修复治理。坚持水资源水生态水环境水灾害统筹治理，全面落实“河长制”，启动泾河干流综合治理项目，加快亭口水库、东庄水库建设，建成红岩河水库、羊毛湾饮水工程；提升城镇污水集中处理能力，推广农村分散式处理技术，持续改善水环境质量。开展第三次全国土地调查，严守耕地红线，提升耕地质量。扎实推进国家森林城市创建工作，推进双照绿林、兴北绿林二期等项目加快建设，全年完成营造林30万亩、绿化道路300公里。严格落实生态环境保护责任，在所有街镇成立环保与村镇管理站，实现网格化管理全覆盖。健全自然资源资产产权制度，严守资源消耗上限、环境质量底线、生态保护红线。调整产业结构、用能结构，推广新能源公交汽车和电动物流替代，加快农村“五改”步伐，从源头上控制污染、减少污染。

坚决打好巩固创文成果攻坚战。全国文明城市是褒奖，更是激励。我们将以永远在路上的韧劲，责任不变、力度不减、考核不松，着力在精细管理上抓提升，消除盲区，打通“最后一公里”，坚决防止脏乱差反弹，确保顺利通过国家卫生城市复审，不断提升城市管理科学化、标准化、精细化、法治化水平，让城市更美丽更有序。着力在以文化人上抓提升，创造性转化咸阳丰富文化资源，提升市民文化中心等公共文化设施服务水平，实施公共数字文化工程，实现县级图书馆、文化馆“两馆”全覆盖，推进建设“书香咸阳”；创新活动载体形式，深化“六进”活动，弘扬“崇德包容、尚法创新”咸阳精神，深耕厚植社会主义核心价值观，激发向上向善社会正能量。着力在深化拓展上抓提升，把文明城市巩固提升融入到各项工作，助推食品安全示范城市等多城同创，支持彬县冲刺全国文明城市，市县同创、城乡联动，让文明之花开遍咸阳大地。

坚决打好优化营商环境攻坚战。实施营商环境提升年“10+3”行动计划，增强投资发展环境竞争力。持续深化“放管服”改革，再梳理公布一批“最多跑一次”改革事项，推广相对集中行政许可权改革试点经验，全面运行“三级联网、四级联动”政务服务新模式。深化商事制度改革，推进“多证合一、一照一码”登记制度改革以及“证照”分离改革试点，深入推进“双随机一公开”事中事后监管，扎实开展网络市场监管与服务示范区创建工作。进一步降低企业运行成本，严格落实减税降费政策，扩大直供电交易范围，探索实施直供气、增量配电等试点，对重大项目、重点企业一事一议，实行更多优惠，打造生产要素洼地。构建亲清政商关系，消除隐性壁垒，帮助解决发展难题，繁荣壮大民营经济。

各位代表，我们将下定攻坚的决心，拿出攻坚的举措，务求打赢打好“四场攻坚战”，向广大群众交一份满意的成绩单！

（七）保障改善民生，着力增强群众获得感

不断提升公共服务水平。推进义务教育优质均衡发展，实施第三期学前教育三年行动计划、高中阶段教育普及攻坚计划，促进职业教育内涵式发展。持之以恒落实社会事业基础设施建设规划，开工建设新渭城中学，2019年建成彩虹第二中学，今年秋季英才学校开学招生。纠正和防止幼儿园教育小学化，解决婴幼儿照护及中小学生课外负担重、“择校热”、“大班额”等问题，严禁调减体育、美术、音乐等课程。全面加强教师队伍建设，加大农村地区教师配备力度，扩大赴先进地区交流培训规模，提高教师地位待遇，努力办人民满意教育。完成二轮市县志书编纂任务。支持文艺精品创作。推进“健康咸阳”建设，持续深化综合医改，健全完善分级诊疗、现代医院管理、药品供应保障和综合监管等基本制度，大力发展智慧医疗。加快市第一人民医院门急诊综合楼、市中心医院医技住院综合楼等项目建设，谋划成立市儿童医院。实施基层卫生能力提升三年计划，加大全科医生招聘培养力度，推广家庭医生签约服务，持续加大“看病难”“看病贵”问题解决力度。支持社会办医，推进医养结合。倡导健康文明生活方式，有效预防重大疾病发生。开展全国食品安全示范城市创建工作，保障群众舌尖上的安全。

织密织牢社会保障网。实施更加积极的就业政策，加大对高科技人才、高校毕业生、返乡农民工创新创业的支持力度，以创业带就业促增收。推进养老和医疗保险制度改革，稳步推进社会保险扩面提标，确保社保基金安全运行、保值增值。提升城乡低保规范化管理水平，确保做到应保尽保、应退尽退。深化居家养老服务试点，鼓励支持社会资本发展养老服务，关爱空巢老人，让老年人享受更加美好的生活。坚持“房子是用来住的，不是用来炒的”定位，推进建立多主体供给、多渠道保障、租购并举的住房制度。把棚改与提升城市品质、改善民生结合起来，投资44.34亿元，建设棚户区安置房1.4万套。

全力促进社会和谐稳定。全面实施“七五”普法规划，扎实推进依法治市。深化平安咸阳建设，完善立体化社会治安防控体系，扎实开展扫黑除恶专项斗争，依法严厉打击“黄赌毒”、电信诈骗以及传销等违法犯罪活动。突出源头治本，严格落实领导包抓重点信访案件制度，加大矛盾纠纷排查化解力度。加强应急保障体系建设，提升防灾减灾能力。深化安全生产领域改革，推进安全生产双重预防机制建设试点，加强智慧安监应用，开展重点行业大排查大整治，坚决遏制重特大事故发生。重视做好民族宗教工作，促进民族团结、宗教和谐。

各位代表！人民群众对美好生活的向往就是我们的奋斗目标。新的一年，我们将突出扶贫解困、生态治理、交通出行、宜居环境、文化体育、健康保障和教育惠民等重点领域，投资120亿元，继续办好30件惠民便民实事。一年接着一年干，一件事情接着一件事情办，让群众生活年年有新变化，让百姓日子越过越舒心。

（八）围绕建设人民满意政府，着力推进政府治理能力现代化

新时代要有新作为、新气象。市政府将深入学习贯彻党的十九大精神，持之以恒建设学习型、法治型、服务型、务实型、廉洁型政府，不断提升政府治理能力现代化水平。

一是大学习，提升履职能力。按照“抓首要、大学习、促发展”要求，扎实开展“不忘初心、牢记使命”主题教育，不断把党的十九大精神学习贯彻引向深入，用习近平新时代中国特色社会主义思想武装头脑、指导实践、推动工作。围绕做到“五个过硬”、提升“八个本领”，开展大学习大培训大调研，加大干部赴先进地区交流培训力度，不断提升广大公务人员的履职服务能力。

二是严法治，推进依法行政。弘扬宪法精神，增强法制观念，严格依照宪法法律行使职权、履行职责、开展工作。自觉接受市人大及其常委会的法律监督和市政协的民主监督，主动接受社会监督和舆论监督，认真听取民主党派、工商联、无党派人士和各人民团体的意见，抓好人大代表建议、政协委员提案办理工作。加强市县政府法治队伍建设，强化对各级政府及其部门决策和规范性文件的合法性审查，积极推进地方立法。深化综合执法体制改革，严格执法监督，促进行政执法规范化。进一步扩大政务公开，扩大和保障人民群众知情权、参与权、表达权和监督权。

三是转作风，狠抓工作落实。践行“三严三实”，弘扬“马上就办、马上就做”精神，求真求实、低调务实，每项工作做到责任严明、督查有力、奖罚分明，抓铁有痕、踏石留印、务求实效。巩固深化“行政效能提升年”活动，传导压力、形成震慑、建立长效，坚决整肃庸政懒政怠政行为，坚决整治不作为慢作为乱作为问题。严格落实“三项机制”，让改革者、实干者、锐意进取者放下思想包袱，甩开膀子大干，巩固心齐气顺劲足的良好局面。

四是守纪律，从严正风肃纪。始终把纪律和规矩挺在前面，以上率下，坚决贯彻中央八项规定《实施细则》、省委《实施办法》精神，以及市委《具体要求》。驰而不息纠正“四风”，坚决反对形式主义、官僚主义，坚决防止“四风”反弹回潮。厉行节约，反对浪费，加大审计监督力度，确保财政资金每一分钱用在刀刃上。坚持惩治腐败无禁区、全覆盖、零容忍，紧盯征地拆迁、脱贫攻坚、惠民利民等重点领域，既打虎又拍蝇，既治标更治本，促进干部清正、政府清廉、政治清明。

各位代表，幸福都是奋斗出来的。让我们紧密团结在以习近平同志为核心的党中央周围，在省委、省政府和市委的坚强领导下，不忘初心、牢记使命，开拓创新、勇于拼搏，奋力谱写追赶超越新篇章，为建设富强人文健康新咸阳作出新的更大贡献！
